# “East” in Europe—Health Dimension through the Lens of the UK *Daily Mail* and Statistical Facts

**DOI:** 10.3390/ijerph18073705

**Published:** 2021-04-01

**Authors:** Izabella Lecka, Viktoriya Pantyley, Liudmila Fakeyeva, Alexandrina Cruceanu

**Affiliations:** 1 Centre for Health Care Management, Faculty of Management, University of Warsaw, 02-678 Warsaw, Poland; 2 Institute of Socio-Economic Geography and Spatial Management, Maria Curie-Sklodowska University in Lublin, 20-031 Lublin, Poland; 3 University of Edinburgh Business School, Edinburgh EH8 9JS, UK; 4 Faculty of History and Geography Ştefan cel Mare, University of Suceava, 720229 Suceava, Romania; alexandrina.cruceanu@gmail.com

**Keywords:** geography of health, health care management, UK, Eastern Europe, Poland, Romania, Belarus, Ukraine, Brexit, newspapers, tabloid, immigration

## Abstract

The study concerns the relationship between health and geopolitics in the United Kingdom (UK). To demonstrate this relationship, we examined the subject and tone of articles published in the popular media (on the example of tabloid the *Daily Mail*) in 2006–2020 concerning health and medical care, and the health and health care practice of Eastern European immigrants belonging to and not belonging to the European Union (EU). There was an increase in media criticism of the behaviour of immigrants in the years 2014–2017, in the period around the referendum in favour of the UK leaving the EU (Brexit). Attention was drawn to the media’s use of a Belief in a Zero-Sum Game (BZSG) narrative at that time. On both sides, “hosts” and the “guests”, a progressive anomy process was observed, degrading the behaviour of individuals and social groups.

## 1. Introduction

With the COVID-19 pandemic dominating the global biopolitical situation, many other links between health and geopolitics remain in the shadows. In the recent past, clear geopolitical concerns about health and migration were noticeable in the world in the 1990s with the AIDS epidemic [1]. In the following decade, similar concerns could be seen in Europe due to the increasing wave of migration from outside the EU, as well as the migration from the poorer to the richer countries within the EU following the 2008 economic crisis. These concerns particularly regarded the effects on health and medical care in the old EU countries after the enlargement in 2004 (Cyprus, Czechia, Estonia, Hungary, Latvia, Lithuania, Malta, Poland, Slovakia, Slovenia), in 2007 (Romania, Bulgaria) and 2013 (Croatia). The country most affected by migrations from the new EU members was the United Kingdom. As a result of a massive migration wave, there was an increase in media criticism of immigrants and their behaviors, which emerged during the UK’s exit from the EU (Brexit) (Appendix A).

Our research was driven by curiosity, to see to what extent the unwelcomeness of the British community towards immigrants from Central and Eastern Europe (CEE) (or more broadly the so-called East) in terms of health and healthcare coincided with the message of the easily accessible media. Besides, there were several questions we were interested in. What were the main points of the discussion? Does the order of appearance of narrative frames confirm the scheme that first come the subjects of infectious disease threats, and then the concerns regarding health care resources? Does the media narrative on health aspects correspond to the actual facts? Finally, is it true that the populist-conservative media maintains the view that “Eastern Europeans are still ‘not-quite-white’” [2] or that they are “a second class EU citizens” [3] or that they are “East European yet not fully European” [4], using the health aspects that underpin human rights.

We examined the subject and tone of articles published in the popular media from the example of the most popular tabloid in the UK with the greatest audience reach (the *Daily Mail*) in 2006–2020 and concerning health and medical care, and the health and health care practice of Eastern European (EE) immigrants. The information presented in the *Daily Mail* was compared to official health statistics for the most mentioned countries both belonging to and not belonging to the EU—Poland, Ukraine, Belarus and Romania—to analyse the gap between the popular media image and the real situation in the mentioned countries. The fear of the East, deeply rooted in the UK and also noticeable in other countries, and the problems still associated with infectious diseases is essentially reflected by facts. However, the East is not a monolith. Each country is struggling in different conditions with similar problems, but is also successful in many areas, as our analysis shows.

Following the connection between health and geopolitics is not only justified, but is also increasingly needed in a globalised world. Understanding this relationship is necessary for societies and countries to be able to react appropriately and in a timely manner, preventing short- and long-term issues.

## 2. Informational Background of the Research

Health must always be considered in a geopolitical context [5,6,7,8,9,10,11,12]. First, the geographical conditions determine the emergence and spread of specific pathogens causing infectious diseases, followed by lifestyle and diverse socio-economic conditions that have particular importance in preventing and reducing the effects of infectious and non-infectious diseases [13]. Politics, both local and international, have an impact on the scope of healthcare, as well as the direction and pace of possible changes in public health. More often than not, it is implemented in a very heterogeneous environment, making the result difficult to predict.

The most tangible evidence of the relation between health and politics is the creation of The World Health Organization (WHO) and its predecessor, The Health Organization of the League of Nations, in the wake of World War I. Many researchers have shown that the international dimension of public health is permeated with colonial, Cold War and contemporary neoliberal rhetoric [14,15,16,17,18,19,20]. Meanwhile, health needs can be physical, psychological, social and financial in their nature. They should be considered not on the individual level, but in a wider context. Then the clash of political views immediately becomes evident, and the problems take on new meanings other than health [13,21]. It should be noted that moods of the so-called public opinion in a threatening situation quite clearly fluctuates depending on the implemented state policy and its effectiveness, but they are also moderated by official media and social media.

In the context of health and migration, the authors of “Geopolitical determinants of Health” [12] (p. 309) warn: “The public and political perception of migrants remains a kaleidoscope of conflicting views. This is particularly true with the rhetoric in Europe with populism offering superfluous answers. In this climate, state-sponsored hate threatens to normalize discrimination against the minority groups. Xenophobic slogans at nationalist marches across many countries and sweeping crackdowns on minority communities showed how the open advocacy of intolerance is increasing”. Their views are shared by other researchers [2,4,22,23,24].

Nowadays an increasing number of researchers are concerned with the populist and nationalist movements that have frequently shaped policy and social responses to COVID-19. During the novel coronavirus pandemic, nationalists tend towards national security policies, close to the phrase “the nation’s interests prevail”; globalists tend to lean towards moderate policies with the faith in the recommendations of the scientific community. The former are largely conservative country leaders, and populists, many of whom uphold racism, while the latter are medical practitioners, intellectuals and philanthropic entrepreneurs. The middle class has an ambivalent role, tending more to the globalist views with a minority belonging to nationalists [25,26].

In our text, we focus on the health aspect in the context of the influx of immigrants, which is undoubtedly a difficult situation, as the host country may justly feel used. National health expenditure will finance not only the needs of its own citizens, but also the health of foreigners. The situation is different in the case of, for example, employment of people who acquired their education or qualifications at the expense of their country of origin. Their work in the host country is often appreciated, they are in demand on the labour market, and they bring profit to the host country without unnecessary expenditure. There are scientific papers confirming that “knowledge-based, highly skilled workers are actively sought out and recruited by governments for their expertise and technical capital” [27,28] (p. 35). However, the labour market is subject to laws other than health protection, and we cannot directly translate the results of researchers’ work on the use of foreign labour into other aspects of the economic and social life of the host countries.

Our work is closer to considering what is happening in individual European countries during the unexpected COVID-19 pandemic, which has raised the profile of geopolitical concerns about health and migration which were noticeable decades ago (e.g., AIDS, TB in the 1990s) [1]. Later on, this developed into the desire to get rid of immigrants in countries with the highest migration inflows like the UK and their supposed pressure on the health systems [21]. In practice, the migration to the UK from the Central and Eastern European states and from southern Europe (in the wake of the 2008 economic crisis), among others, was one of the three major arguments in the Leave vote at the 2016 referendum [29,30]. Following the protracted confrontation of supporters and opponents of UK membership in the Community, the country ultimately left the EU on 31 January 2020. 

In the UK, in addition to the general feelings of hostility often expressed towards foreigners coming in search of work, there was a growing aversion to the rapidly growing group of migrants from Central and Eastern Europe, including apparently the rapidly growing number of Poles [4,22,31,32,33]. The health concerns frequently raised initially included the supposed growing threat of transmitting infectious diseases, especially tuberculosis. Soon the dominating argument was the excessive use of free medical care by the arriving foreigners. Migrants from Eastern Europe reportedly had higher rates of chronic diseases, including diabetes and cardiovascular disease. Non-specific or somatising presentations as a result of psychosocial distress also prevailed [21].

David Cameron kept his 2015 pre-election promise and decided to hold a referendum on the country’s membership in the European Union as well as precise guidelines in health care, such as reciprocal health care charges for foreign nationals [22]. The referendum took place on Thursday 23 June 2016; 52% of the population voted Leave and 48% voted Remain [34]. Regarding health and healthcare, there were soon voices among supporters of staying in the EU about the disastrous impact of this decision on the financing and functioning of the UK National Health Service (NHS). A few months later, leaflets saying 75% of Health Leaders think Brexit will have a negative impact on the NHS, and none of the remaining 25% believe it will have very positive effects, were distributed all over the country [35].

It should be noted that in media article headlines, the word “fear” is frequently followed by the term “East”. For the British, this “disturbing” East is represented by the countries of Central and Eastern Europe [4,22,31,36]. British sociologists also point to a feedback loop in this situation. The aggressive attitude towards migration from Eastern Europe (widely reported in the media and police statistics) “…combined with the frustration and resentment that festers with entrenched marginalisation (as suggested by the ethnic penalty research), contributes to a more generalised climate of fear, anxiety, and suspicion amongst Eastern Europeans” [2].

While global openness has become the widespread official ideal worldwide, prejudices against immigrants are still common among the general public, especially in countries with strong feelings of national identity [37]. Migrant communities are often presented as “contagious” and undesirable (regardless of the political system), and national public health activities sometimes blur the differences between liberal–democratic and authoritarian states [21,22,38,39,40]. Research by British sociologists suggest (‘Hungarian and Romanian migrant workers in the UK: Racism without racial difference?’ ESRC grant number RES-000-22-3358, 2009–2011) that different forms of racism and xenophobia still present in British institutions are transmitted through political discourse [2]. This is also confirmed by other researchers [3,22].

Fortunately, politics is not a zero-sum action, and even if that were the case, a fixed sum of the game, particularly a zero sum, does not imply its fairness. Belief in the Zero-Sum Game (BZSG) is based on the implicit assumption of resource constraints, so whenever someone gets a benefit, others have to lose. In economic terms, the zero-sum game with regard to the healthcare system seems to be a tempting argument to apply, especially by populist politicians. However, because BZSG is both an individual and a social phenomenon, the effect of an individual BZSG may be different in societies with different levels of BZSG at the country level. A study of the example in 35 countries proved that the perceived antagonism in social relations has a negative effect on life satisfaction. However, the relationship between individual BZSG and the negative impact on life satisfaction was less noticeable in societies with higher national BZSG [41]. Thus, a study of the example of a fairly ethnically and religiously homogeneous society (Poland) showed that patriotism is positively related to the willingness to accept migrants through a negative relationship with prejudices through decreasing BZSG. On the other hand, nationalism is negatively associated with the willingness to accept refugees through prejudices resulting from the growing zero-sum thinking [42]. In order for a successful policy to arise, the mentality has to shift from a zero to a positive sum. However, this is a long process.

British sociologists came to the disturbing conclusion that racist and xenophobic behavior in the UK towards people from Eastern Europe increased after the Brexit vote. Their study proves that this problem is nothing new and has little to do with the EU structures [2]. But it should be emphasised that this is a rather particular problem. For the first time in Europe an escalating societal crisis led to a rescaling of the state. Mass migration to the UK was legitimised by international legislation, while the state policy presumably should have limited the potential negative effects of this transnational process. No surprise then that the “fourth power”, i.e., the media, took such an active part in the situation. They suggested to the politicians the ideas that could be eagerly accepted by the public. In that case, “British newspapers rearticulated the changing relations of scale between the state and the EU to legitimize differential levels of EU citizenship” [3] (p. 411).

## 3. Materials and Methods

Studies suggest that migration, the behaviours in British society that eventually led to the UK’s exit from the European Union, and the media influence were all inter-connected [2,3,22]. However, none of the media focused solely on health issues, although they were regularly brought up. Studies considering just health and the use of the NHS by Eastern Europeans in the UK are rare to find in the literature [43].

Generally, most UK newspapers expressed constant skepticism of the EU. Admittedly, this group was shaped by political orientation and can be qualitatively divided into broadsheets (more profound newspapers) and tabloids. Still, their message to the readers contained many common features [44]. Media coverage revolved around negative themes, and “the terms asylum seekers, immigrant and migrant were used almost as synonyms”. The left-leaning newspapers and tabloids used “more humanized explanation but were evasive and unspecific when immigration was central to political debates” [3] (p. 413).

The most popular newspapers in the UK are the left-wing broadsheet *The Guardian* and the left-wing tabloid *Daily Mirror*/*Sunday Mirror*, and the right-wing broadsheet The Times and the tabloid the *Daily Mail/Mail on Sunday*. A study by U.S. researchers suggests that in the representations of Polish Migrant Workers, only The Guardian stood out with their pro-immigration attitude. Other media expressed varied and conflicting views [3].

Our aim was not to compare the messages in different media, but to consider the most popular (i.e., with the greatest audience reach) narrative. That is why we have reviewed the texts that appeared on the MailOnline website (containing published texts of the highest-circulated daily newspapers in the UK: *Mail on Sunday* and the *Daily Mail*). The *Mail on Sunday* was the most-read Sunday newspaper in 2019. Together, the paper and digital versions of The Mail on Sunday enjoyed over 33 million monthly users (second only to *The Sun*). The main target audience is lower-middle-class British women, and The *Daily Mail* was the first newspaper in the UK to start publishing articles targeted at the female audience [45]. Of course, it would be wrong to just categorise it as a women’s newspaper (in fact, the sheer number of its users contradicts such an idea). The *Daily Mail* has won National Press Awards on eight occasions since 1995, including several titles of the National Newspaper of the Year; most recently in 2019, which “was a sensational year for the *Daily Mail* with unforgettable scoops, campaigns and front-page splashes” [46,47]. Considering the populist use of these scoops and campaigns by the tabloid, we decided to particularly focus our analysis on this medium. The fact that these are exactly populist-conservative media was a coincidence, not a choice of the researchers. However, the studies of the aforementioned researchers show that the British media very often take on a populist or populist-conservative perspective in their coverage of immigration-related issues.

Moreover, in the study we leaned towards the point expressed by Light and Young [4] (p. 286) that “the tabloid papers were far more active in shaping and responding to popular discourses […] they played more of a leading role in provoking debate”. This is also true of the health debate. *The European Journal of Communication* [23] confirms this, emphasising that tabloids are the main venue for the everyday narrative on various subjects including crime and health and that they influence human behaviour [4,48].

In our research we adopted a critical perspective through the following steps:

(1) the problem was identified on the basis of literature;
(2) sources of popular information, easily accessible to the reader, were searched for (publications from MailOnLine); articles were searched according to national terms/name of the region and countries of the region (Eastern Europe, Poland, Romania, Ukraine, Belarus) and the term “health” or country name and “health care”, according to Boolean logic. Papers that met the following criteria were included: (i) the content concerned the selected countries/region; (ii) health and healthcare; (iii) and was related to the situation in the UK.
  The texts that (i) dealt only with the situation in the selected countries with no relation to the situation in the UK; (ii) *de facto* did not touch the subject of health or medical care (for instance, health was used only in a metaphor); (iii) contained only one sentence regarding health; (iv) concerned the health/injuries of professional athletes, were excluded.
(3) analysing each of the selected articles was supported by: (a) Critical Discourse Analysis where languages play a vital role in describing the social reality within intertextual and socio-political contexts [49]; (b) frame theory from the Scheufele and Tewksbury perspective [50], which describes the frames used by the media as macro-constructs, necessary to reduce the complexity of the issues and add meaning to the information presented. We considered a generic frame [51], which we defined using two criteria: (1) the titles of articles, and (2) the content, bearing in mind that it is typical of tabloids to seek sensation in order to increase their readership [23,52].
(4) then, through the analysis of data included in statistical reports and scientific sources (not characterized by any emotional tone), a comparison between the media narrative and the facts was made according to the key issues selected in the previous step in terms of the health situation in the most-mentioned Eastern European countries in the *Daily Mail* during the study period. There were two EU countries (Poland and Romania, whose citizens have most frequently migrated to the UK in recent years) and two neighboring countries to the east of the EU border, Belarus and Ukraine.

The analysis of the articles was carried out from the narrative perspective as qualitative research requires reflection and reflexivity [53]. Even if individual reality is a phenomenon, social reality is a construct shaped by narrative forms. Narratives are not just an arrangement of information. They are, in essence, a construction of reality. Narration is a conceptual framework for understanding human decisions [54]. Any media text requires a narrative structure (a frame) to organise its discourse. Generally, attractive news pieces are based on narrative conventions that offer an explanation about who is doing what, and with what purpose [51]. News items prepared especially for non-inquisitive readers also suggest an interpretation of the facts and a solution to the problem.

In the Discussion, we use a “critical friend” method [55], where the role of this critical friend is “not to ‘agree’ or achieve consensus, but to encourage reflexivity by challenging each other’s construction of knowledge” [56] (p. 508).

We did not go into the media articles authors’ motives, which govern their views, because we adopted the well-known humanistic perspective that the social world is a collection of stories from which we choose particular ones [57].

We tried to avoid moral assessments of the presented problems, as did for, e.g., Simon Cross [23], assuming that anomy may be characteristic of both the British and of economic immigrants from CEE countries. It may be caused by both the mass influx of “foreigners” as part of the EU enlargement and, in the case of the CEE countries’ citizens, by double adaptive stress resulting from the dramatic political and economic transformation after the collapse of the Eastern Bloc, and the subsequent participation in the European Community and, more broadly, in the globalization processes. Anomy causes degraded adaptive reactions that may cause a departure from the current values and the disappearance of social norms [58,59].

## 4. Results

A total of 37 articles published in 2006–2020 were analysed, moreover, 34 of them appeared between 2014 and 2020 (see Appendix A). The articles from 2020 were omitted (with one exception) in the analysis, as many of them concerned the COVID-19 epidemic and did not relate to the main goal of the study. There were many more texts concerning immigrants. They touched upon such subjects as cultural differences, higher competition at work and willingness to take jobs that are underpaid or below the qualification, as well as demographic dominance in some areas, and crime. The issue of foreigners on the job market was discussed particularly often. Instead, we concentrated on the content regarding health. That is why the number of articles is not high, but they are important for public discourse.

The content of these texts can be divided into 4 narrative frames by their main subject (in one case, the article is included in the three frames (A:14)) (see Appendix A):

A. Diseases (10 art.); keyword: measles, HIV/AIDS, TB, Hepatitis B and C, vaccination;
B. Health Care Resources (16 art.); keyword: health care, health care workers, pressure on health resources, health tourism;
C. Maternity (5 art.); keyword: baby boom, children, mothers;
D. Style of life (8 art.); keyword: healthy style of life, death rate, alcohol, stress, women.

Most of the texts concerned Poles (16), Romanians (12), followed by Ukrainians (11), as well as Eastern European citizens in general (7), and Belarusians (5), with sporadic mentions of Czechs, Slovaks, and Lithuanians.

The coverage by the *Daily Mail* of these main issues is divided by country and by region among Eastern European members of EU as follows: Poland—FRAME A (1 art.). B (12 art.), C (4 art.), D (3 art.); Romania—FRAME A (3 art.), B. (7 art.), D (2 art.). In the case of other Eastern Europeans: Ukraine—FRAME A (4 art.), C (2 art.), D (5 art.) and Belarus—FRAME A (2 art.), C (3 art.), D (2 art.).

Basically, the citizens of EE members of the EU were mostly brought up in relation to health care resources (Frame B), while the citizens of other EE countries, in relation to diseases (mainly infectious) (Frame A). Publication with “Eastern Europeans” as a research entity consists of 7 texts: FRAME A (3 art.), B (3 art.), C (1 art.) and D. (2 art.). Almost half of the articles concerned women, children, pregnancy, and childbirth, presumably confirming the pro-female profile of the tabloid.

Infectious diseases were a trending topic in the 2018–2019 articles due to the outbreak of measles in Europe. However, the greatest number of diseases (HIV, TB, Hepatitis B and C) was brought up in a 2015 text on immigrants, i.e., in the year before the Brexit referendum. In the case of Ukraine and Belarus, these stories were also touched upon other media since the 1990s (see BBC Monitoring Former Soviet Union).

At the time of the 2004 EU enlargement, the first texts that appeared in the tabloid concerned the issues of maternity among immigrants. However, the greatest number of texts on the issues of health and medical care among Eastern Europeans appeared in 2014–2017 (21 art.) Frame B was dominating among the themes (13), while other issues were covered less frequently: A (3), C (2), and D (3). Numbers of articles in Frame B contain politically engaged texts (not based on scientifically proven data).

The titles of the articles considered were analysed using Word Frequency Counter. Among others, the following words assessing the situation came out: “alarm”, “against”, “violence”, “unprotected”, “unhealthy”, “unacceptable”, “suddenly”, “struggle”, “stress”, “slavery”, “shocking”, “scandal”, “unacceptably”, “screw system”, “horrific”, “complete chaos”, highlighting the articles’ shocking content.

**Figure 1:**
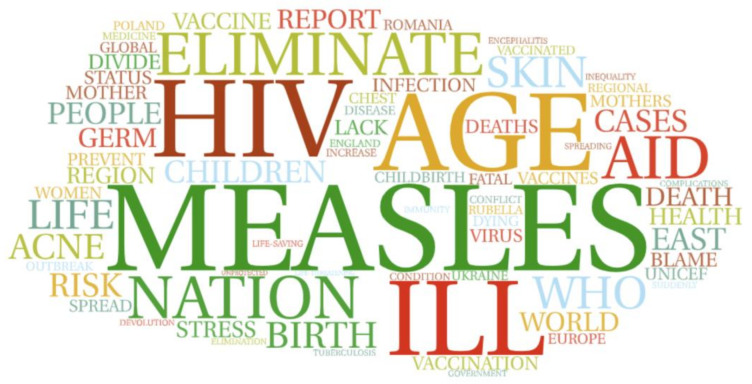
The most frequent words in the health dimension used in the *Daily Mail* articles concerning Eastern Europe. Source: authors’ elaboration based on WorldClouds.com.

The evolution of the narrative can be observed on the basis of the articles’ titles (Figure 1). It ranges from quantitative issues (“5000 Polish babies born in UK every year”) to system pressure (“My shocking encounters with health tourists who exploit NHS” and “Immigration is placing a strain on the NHS…”) (in chronological order) (A: 37, 22, 14).

Six other texts analysed (16%) had a message of approval, although the Word Frequency Counter did not detect words with a positive connotation to be as strong as the above-mentioned negative ones. Of the five texts regarding lifestyle, four portrayed Eastern European citizens (at least in most countries) in a positive light, although the texts considered different behavior in the countries of origin. The articles used the data of such well-known global organisations as the WHO, the European Institute for Crime Prevention and Control, and Save the Children. The 2011 text even mentioned British immigration to Poland: “Lifestyle ‘is better in Poland than Britain’: Less crime and violence—and it’s cheaper too” (A: 35). Yet, we can notice the tone of disbelief in the titles of articles: “Mothers in the UK are almost FOUR TIMES more likely to die in childbirth than those from Poland and Belarus”, “US maternal mortality rate is worse than Iran and Ukraine”, or that of reproach or regret: “Why ARE women in the UK twice as likely to die in childbirth as their Polish peers?” (A: 3, 23, 29)

Most of these texts were original, written by journalists, but five of them are compilations for the *Daily Mail* of the information from the Associated Press and Reuters agencies. Generally, the latter concerned broader studies about the world and the region, often based on newly published maps and reports.

Regarding the journalists, we can distinguish texts written by people specialising in medical matters and using statistical data and scientific research in their articles, even if they were ultimately loosely interpreted (A). We can also see among the authors people specialising in presenting the opinions of politicians, and from their statements emerge their political preferences, not hard data (A: 12, 15, 20–22, 27–28, 37, 44). There is also a short article symbolically signed by no fewer than four authors (A:28). The names of some of them appear only in publications preceding the Brexit referendum (A).

We immediately came across a certain inaccuracy due to the everyday media understanding of geographical categories. Eastern Europe is sometimes understood as all the countries of the former Eastern Bloc (A: 34) or as new EU countries in Eastern Europe (A: 14, 28) or with little precision (A:20), as the text refers to persons who would show a document in a hospital entitling them to free medical care, and the rights of EU citizens or non-EU citizens were different during the years considered and the text for both groups is incorrect. “East European members of EU” are rarely clearly defined (A: 25).

In order to understand health inequalities in various aspects, including socio-economic development and living standards, much has been done in 2015–2017 as part of the EURO-HEALTHY (Shaping EUROpean Policies to promote HEALTH equitY) project, the results of which are available online [60,61]. The Synthetic Population Health Index of 0 to 100% score (as a measure of economic, social and security dimension, education, demographic change, lifestyle and health behaviour, physical environment, built environment, road safety, healthcare resources, expenditure and health performance) obviously shows differentiation between nations. However, it is often even higher at the level of national administrative units. This index for Poland is roughly 75% for the whole country, compared with the stretched values for the UK between 75–85% (capital city with the best score). In general, however, EU countries in Eastern Europe rank lower in the index (with the exception of the Czech Republic), with Romania and Bulgaria ranking the lowest, and there are more disparities within the country (50–70%) [61] (p. 55). This corresponds with further research by Santana et al. (2020) where counties’ level of development and GDP is highly related to Population Health Index (the mean difference between LD and MD European regions is the highest in healthcare performance, housing conditions and waste management) [62] (p. 10), as well as “LD regions present significantly worse population health scores in all dimensions of health determinants, compared with those presented by MD regions” [62] (p. 14).

## 5. Discussion

Most articles in the *Daily Mail* (version MailOnline) concerned Eastern Europe or Poland, and to a lesser extent Romania and other EU members after 2004. This is understandable, because immediately after Poland became an EU member, Great Britain opened its borders to employees from this country, and only in 2014 (after 7 transition years) to employees from Romania and Bulgaria. EU immigration rose sharply in the years preceding the EU Brexit referendum in June 2016 but has declined since [63]. After all, in 2019 there were twice as many Poles in the UK (900 thousand, and peaking at 1021 thousand people in 2017), as Romanians (450 thousand, 2nd place among new EU members in the UK). For comparison Lithuanians (168 thousand) 7th place among new EU members in the UK and Bulgarians (128 thousand) 10th [63]). Half a million Poles were in the UK already in 2008 (4 years after the opening of the markets). Among Romanians the biggest annual increase of 95,000 occurred between 2015 and 2016 [64].

In the end, the migration traffic from both countries was quite similar, reaching 2.6–2.7% of the population of the home country in the case of Poland, and 2.3% for Romania. However, the number of texts concerning Poles in the analysed media was significantly higher than that of Romanians (even if the texts concerning the Romanian Roma community were taken into account). This is understandable, because ultimately there were more Poles (23% of the total number of migrants from the EU to the UK). They began to actively participate in the labour market earlier, and soon they were more and more actively enforcing their rights with regard to medical care, generally available free of charge for people living in the UK. However, since Brexit, the desire to stay in the UK among Poles has decreased. In June 2020, for several nationalities there have been more applicants to the EU settlement scheme (EUSS) than the upper limit of Office for National Statistics (ONS) population estimate (for ex. Romanians, Bulgarians, South Europeans) but Poles are the least represented in this group [63].

It should be noted, however, that the overall media coverage in the UK press varies considerably depending on the nationality of the EE representatives. Some nationalities (such as Poles) are the subject of both positive and negative news, some (such as Romanians) are mostly negative, others (such as Czechs and Estonians) enjoy media attention only sporadically [4,22].

Due to the different areas of interest and potential conflict between the interests of the UK and the representatives of Eastern European countries, the articles addressed to some extent different issues. In relation to the countries bordering the EU in the East, public health issues dominated (Frame A), including the increasing number of patients with infectious diseases over the years, mainly tuberculosis (TB) and HIV infection, and in 2017–2019 the risk of measles recurrence and the dangers that it can cause in the UK. It was questioned whether the extent to which the medical care system in countries such as Belarus, and especially Ukraine, is efficient or not in this difficult situation. It must be admitted that the alarming tone of the statements did not change throughout the entire period.

Health inequity based on official statistical data is clearly observed between studied countries with a visible “East-West” dichotomy in many cases but does not fully correspond with the tabloid’s informational realm.

One of the most mentioned diseases for the Eastern Europe in the FRAME A is tuberculosis. Ukraine and Belarus continue to be among the high priority countries for tuberculosis in the WHO European Region and mentioned in popular media more often than EU member states. Wide coverage of BCG immunisation (97 % for Belarus and 84 % in Ukraine in 2019) [65], has made it possible in the last decade to achieve significant results in reducing the incidence of TB, especially in Belarus. TB prevalence and incidence rates in the Ukraine is still a severe medical and social problem [66]. There are still some alarming figures in the region, e.g., multidrug-resistant TB, which is one of the major public health threats, not only in Belarus or Ukraine, but in most post-Soviet countries [67,68,69]. As a result, Belarus and Ukraine remain as potentially TB dangerous countries for the United Kingdom. To get a British visa for a visit longer than 6 months, citizens of both countries must have a chest x-ray to test for TB at a UK-approved screening clinic [70]. At the same time, tuberculosis incidence rates in Romania are more than twice that in Belarus (66 vs. 29 per 100,000 in the 2019) and comparable to Ukraine, but are disregarded by the popular media [65].

**Figure 2:**
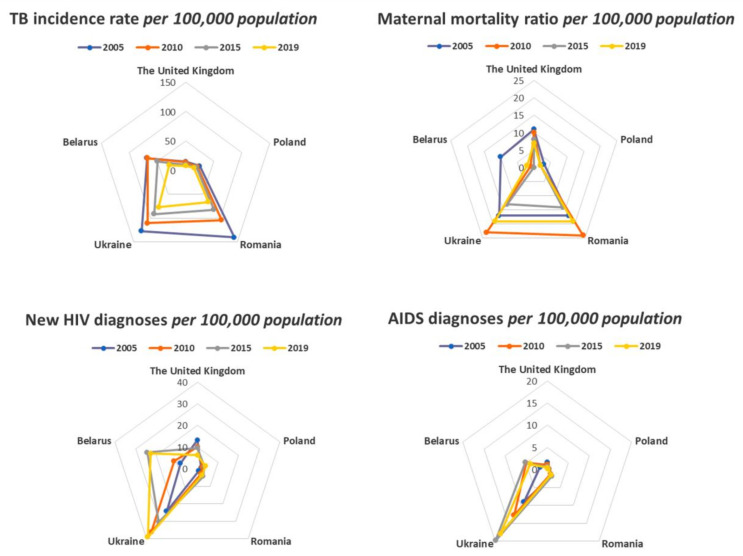
Comparison of the UK and the most mentioned Eastern European countries in the *Daily Mail*, 2005–2019. Source: Own elaboration based on the WHO data [72].

Alarming rates of anti-TB drug resistance are being studied together with increasing incidence rates of HIV infection in Eastern European countries [71]. The clear leader among studied countries by HIV morbidity is Ukraine. Country by the incidence rate at the beginning of the 2000s, was treated as a crisis region of Eastern [66]. Belarus as well has been mentioned among crisis areas in Europe, especially in the 1990s. UK rates for new HIV diagnoses per 100,000 in the early 2000s were much higher than in Poland, Romania and Belarus (Figure 2) [72].

The only topic concerning infectious diseases that connected all the countries in a similar way was measles. According to the WHO, measles emerged worldwide in 2019 reaching the highest number of reported cases in 23 years. The highest numbers of cases were recorded in 2018 and 2019, both in the east and in the west and south of Europe (A: 4–8). There was a widespread consensus that this was due to the lack of vaccination of an appropriate percentage of people in individual countries. Eastern European countries of the EU and non-EU countries were in a relatively better situation. As a legacy of socialism, there is still considerable vaccination coverage of the population, especially in middle and older age brackets [73]. The growing group of vaccine sceptics has only emerged now in the era of migration to a much more sceptical Western Europe. Moreover, numerous economic and political problems of Ukraine resulted in the postponement of measles vaccination a few years ago [74]. Sudden outbreaks of the disease also appeared in Poland, which is estimated to host 2 million economic immigrants from Ukraine (A:7), as reported in 2019 by the *Daily Mail*.

Regarding the European Union countries for which the name A8 was introduced in the UK (all 2004 EU enlargement countries except Malta and Cyprus), the topics mostly concerned women’s fertility, children’s health, medical tourism, the quality of health care in A8 countries, employment of medical personnel from Poland, as well as the broadly understood use of UK National Health Service (NHS) funds by immigrants (Frame B, C).

The hottest and most threaded topic was the baby boom among Polish women living in the UK and the related costs of protecting their children’s health and the use of National Health System resources (Frames B, C). An alarmist article from 2006 reported that 5000 Polish children are born annually in the UK (A:37), and Polish women in exile give birth to children much more willingly than at home. This theme in the 2011 article takes on a slightly different tone, as the example of one of the hospitals (Ealing Hospital West London) showed that 80% of children born there had mothers of 104 nationalities, including Polish mothers in the 2nd place after Indian mothers, followed by Sri Lankans, Somalians, Afghans, and Pakistanis. The information contained in numerous comments below the article indicates that this situation is exceptional, because Ealing Hospital is located in the “immigrant” part of London. This problem should not be extrapolated to other hospitals either. However, this situation, even for Polish researchers, is surprising because in Poland women’s fertility has been decreasing successively (to 1.4 in 2020) since the 1980s (and even since the 1950s), despite numerous social campaigns and financial incentives from the government. Meanwhile, in the case of Polish women in the UK, fertility has been at the level of 3.3 in recent years [75]. It seems that despite the fact that the UK is not the country with the highest social benefits, assistance in caring for a child from the age of 2 allows for the possibility to combine work with parenthood, which is definitely more difficult in Poland.

The Office of National Statistics (ONS) officially informs that in 2019, Poland (16,737 live birth), Pakistan and Romania (16,069 live births with a population half the size of Poland’s) are the three most common countries of birth for women born outside the UK who gave birth in England and Wales. The greatest increase occurred in the case of Romanians since 2012. For Polish women, the largest number of children were born in 2015—22,928 [76,77]. The greater fertility rate among non-Britons was associated with financial problems with caring for sick children from the perspective of the *Daily Mail* (A: 14): “THE EXPLOSION FROM EASTERN EUROPE... Figures reveal a 14.3 per cent rise in youngsters born to immigrants being treated in pediatric intensive care since 2004 in England—when former Prime Minister Tony Blair threw open Britain’s borders to the Eastern Bloc”.

Over time, other points were analysed in the field of women’s fertility, namely a certain “level of luxury”. According to the authors (as well as those who commented or “liked” the text), this phenomenon concerned women from Eastern Europe, including, as previously mentioned, women from Poland. This luxury was going to a hospital in Poland to give birth to a child at the expense of the UK NHS, having secured a European Health Insurance Card in the UK. Visitors from the European Economic Area (EEA) are usually covered by agreements under which their home country pays for treatment and vice versa. Although this was legal (in addition A8 nationals of EU coming to the United Kingdom were legally allowed to work, but to do so had to make sure they were registered with the Government’s Worker Registration Scheme. They were entitled to some basic benefits, such as Housing Benefit, Council Tax Benefit and Tax Credits. Only after they have worked legally for at least a 12 month period, without a break of more than 30 days, could they apply for social security benefits such as Jobseeker’s Allowance [78]) for EU citizens who live, work, and pay taxes in the UK, such behavior is represented in the articles as health tourism and linked with medical care for all other migrants, or even described as a “screw system”. The texts were published successively in 2015–2016 and became a fora for statements by politicians from two main political parties. On the one hand, the negative financial aspect was emphasised: “The Committee drew on official figures released earlier this year which show that in 2014/15 £674 million was charged to the UK government for the care of British citizens in EEA countries. But the amount charged for the care of EEA nationals in British hospitals was just £49 million […] Poland charged the government £4.3 million for the medical care of British patients, while the UK charged the Polish Government £1.5 million for the treatment of Polish people in the UK, the Committee was told.” (A: 22). The difference in quotas between Poland and the UK is not surprisingly high in relation to the entire difference (which at the level of whole EU is probably generated by the British pensioners living in Spain and in other countries according to some politicians). The fears of opponents are thus unjustified as the European Health Insurance Card (EHIC) disbursement (Reimbursement up to the amount had the treatment been carried out in the home country [78]) mechanisms result in a higher relative financial burden for EE healthcare systems due to differences in East European and West European healthcare costs [33].

On the other hand, it was pointed out that British health care workers are not border guards and are not entitled to ask about the patient’s ID document. After the Brexit referendum, however, such a regulation (after the Brexit referendum, EU citizens who wish to obtain permanent residence must show Comprehensive Sickness Insurance (CSI) [32]) began to be introduced in the health service from 2017. It was described in the *Daily Mail* as a story of discrimination, using the example of a young woman, a native British woman with a Polish husband’s surname (A:15). This decision was preceded by a series of articles with politicians’ statements pointing to the need to limit benefits (including health benefits) for migrants from Eastern Europe (A: 19–21, 24). Labour party politicians took the position that it was disgraceful.

Research among Polish migrants in the UK confirms however that they “…utilised a variety of sources of medical help, including self-care, private ethnic doctors in the host country, private and public health care systems in the home country and the host country’s public health care system. Preferences have changed over time” [43] (p.111). One might wonder why Polish, Slovak and Lithuanian women wanted to give birth in their home countries if the UK is a richer country with a better standard of living. One answer to this is to be close to the family and get their support. However, the text that appeared in the *Daily Mail* in the same year (not related to the previous ones) indicated much more effective maternity health care in countries like Poland and Belarus (A: 23, 29). The British commentary focused mainly on the difference in age and body weight of British women who give birth (older and stouter). On the other hand, Polish and Slovak women in the comments emphasised that the 24-h delivery system in the UK is much less safe in their opinion than the 3 days in a Polish or a Slovak hospital with a spectrum of anaesthetics. Polish women have greater trust in the Polish health service in the field of childcare, where the indicated specialist is a paediatrician. In the UK it is the General Practitioners (GP). 

The lowest maternity death rates (A: 3, 14, 23, 29) are in Belarus and Poland. Maternity death rate in Belarus showed a tenfold decrease within the last decade [79]. Now, Belarus is among the countries with the lowest indexes in the world about 0–1 per 100,000 live births in 2005–2019 [80]. Poland has almost the same level (2 per 100,000 live births in the 2017) and it is more than 3 times less than in the UK (7 per 100,000 live births in the 2017) (Figure 2). Equally high levels of maternal mortality (19 per 100,000 live births in the 2017) are presented in the Eastern European countries both belonging to (Romania) and not belonging (Ukraine) to the EU.

Another indicator of this assumption is the willingness to employ medical personnel from Poland described in two articles: on paramedics (A: 31) and nurses (A: 32), as they are speaking Polish what is their advantage, in view of the growing number of patients from the same country. In the literature, some authors suggest that investment in advocacy workers may be more efficient than providing more healthcare staff [81]. Still, a spokesman of South Central Ambulance Service said: “We have been carrying out some international recruitment in Poland for paramedics where their qualifications, skills and experience are very similar to our own and meet our own high standards for staff.” [A:31] This whole situation is interesting because the great internationalisation of the medical personnel of the British health service, especially at the lower level, is well known, and the arrival of Polish personnel did not change much here. At the same time, the information contained in the leaflets of the European Movement United Kingdom from 2017 indicated a 92% drop in applications for NHS jobs from EU nurses since the Brexit referendum [35]. Already after the Brexit referendum, news about young Polish medics fighting for material support for the health service with the Polish conservative PiS government appeared on the *Daily Mail* forums (A: 16,17). 

In 2011, a text was also published indicating migration from the UK to Poland, a country less wealthy but safer, cheaper and with a better lifestyle (A: 35). And in the difficult year of 2016, Reuters published an optimistic message in the *Daily Mail* suggesting directly that the apparent increase in living standards of Eastern European members of the EU will certainly reduce the rate of migration from these countries to the UK (A: 25). This was the case, although the reason for stopping migration was probably the Brexit-related decisions concerning immigrants, along with a real improvement in the standard of living in the countries of emigration [64]. When we look at the OECD Better Live Initiative, the data inform us differently, that sometimes life satisfaction in Poland is lower than the average in OECD countries [82], and sometimes higher [83]. This, of course, allows both scientific and media articles to select the data that suit the authors’ concept. Yet, regardless of the data source, life expectancy at birth in Poland is 78 years, two years below the OECD average of 80 years. Life expectancy for women is 82 years, compared with 74 for men. And particularly health is rated as less satisfactory by Poles than on average by society in OECD countries [43].

The dynamics of life expectancy at birth in Belarus over the past 50 years has been characterised by contrasting trends. In the second half of the 1960s, it reached a fairly high level (72.9 years in 1964–69) and then declined until the end of the 1990s (67.9 years in 1999). The exception to this thirty-year decline is a short-term increase of life expectancy at birth in the mid-1980s (72.6 years in 1984–85) [84]. The beginning of this century has been marked by a steady progressive growth of this index, and in 2018 life expectancy at birth for both sexes was 74.5 years. Despite the progress made in increasing the life expectancy of the population, the gender gap remains significant in Belarus as well, amounting to 10.2 years in 2018 for the country as a whole. This gap is mainly due to the high mortality rate among men of 45 to 80 years of age. Within this age interval, the probability of dying for men is much higher than the corresponding probability for women [84,85].

In 2019, the index of life expectancy at birth in Ukraine reached 72.01 years, and the differences in the numbers between the genders for different years, starting from Ukraine’s independence, are rather stable for about 10 years, with the disadvantage for the male gender. The significant disparities in life expectancy between the genders result from the premature mortality of the male population due to cardiovascular diseases, malignant tumors, as well as external causes of death [65].

In 2018, a Death Map of the World was mentioned in the *Daily Mail* (A: 11) showing 10 nations which have the highest probability of men and women dying from a major NCD before they turn 70: Mongolia, Fiji, Kazakhstan, Turkmenistan, Russia, Belarus, Ukraine, Georgia, Kiribati, North Korea. The risk of death before 70 in the non-EU EE countries is 3 times higher than in the UK. As always, the report also points at a much worse health situation among men than among women, not only in Eastern Europe, but also in the UK. Female probability of dying between 30 and 70 years of age from NCD4 (cancer, heart diseases, lung diseases, diabetes) in the EE countries belonging to the EU ranges between 10.2% (Czechia) and 15.6% (Hungary). For men, the figures are between 20% (Czechia) and up to 32% (Bulgaria), compared to the UK (9 and 12.9% respectively) and Germany (8.9 and 15.2%).

The lifestyle of the Eastern European countries outside EU can be characterised as rather unhealthy compare to EU neighbours. This is evidenced by, among other factors, high levels of alcohol consumption. According to the WHO, Belarus, Lithuania, Russia, Romania, Ukraine, Czechia and Slovakia were among the top ten consumers of alcohol in 2008–2010. On the other hand, Global Drugs Survey by independent research company based in London indicated that the UK ranked first. “Adults in the UK get drunk more often than anywhere else in the world”. And this message was also equally reported by the *Daily Mail* [86].

In turn, Polish citizens appeared in the text of the 2015 article (A: 30) on healthy lifestyles as a nation suffering from chronic sleep deficiency. There is a certain coincidence here with the data compiled in the UK by The Migration Observatory. It showed that the largest percentage of people (among all migrants to the UK) who declare coming in search of work or study (84%) belong to the A8 group. Moreover, “unemployed migrants from EU countries were less likely to claim unemployment benefits (16%) than UK born unemployed workers (26%)” [63].

A seemingly light article in the *Daily Mail* (A:1) from 2020 about the problems of a young Ukrainian woman (working in Germany) whose “skin suddenly broke out”, however, ends with unsettling conclusions. It fluctuates around the serious issue of migration-related stress, which can cause serious damage to health. Canadian research on the quality of life and health of working immigrants has shown that skilled immigrants who are working in an occupation that under-utilises their knowledge, skills, and experience suffer from work-related stress, poor quality of life, and finally deteriorated physical and mental health [87]. On the other hand, research in the EU on the example of Ireland, Portugal and Spain showed that these countries developed a theoretical framework of a great range of policies aiming at improving access to healthcare services for immigrants that can inspire other European countries [88].

## 6. Conclusions

In the time of globalization, a problem like the ones we analyse in our article will be repeated constantly. After the end of the COVID-19 pandemic, global migrations are likely to increase and the health problem will not disappear. The need to reconcile the arguments of both migrants and host countries will always require an urgent solution. Understanding the causes of conflicts will create a chance to prevent their occurrence in the future. Better prevention of such issues may limit the chance of an uncontrolled development of intolerance, xenophobia, and aggression accompanying the growing populism in the world. Health, especially in terms of the use of medical care, is a useful thread for populists as it involves “giving” to foreigners (hence apparent loss) and not “taking” (apparent profit). Understanding the relationship between health and politics is necessary for societies and countries to be able to react appropriately and in a timely manner, preventing short- and long-term issues.

The main subjects considered in the UK *Daily Mail* publications can be divided into four frames: Diseases, Health Care Resources, Maternity, and Style of Life. Whereas the stories regarding infectious diseases or the effects of different lifestyles were of an informative and rarely a polemic nature, maternity and healthcare resources became the ground of heated discussions. We are witnessing a politicization of health, with moods ranging from fear of disease to fear of undesirable healthcare.

The fear of the East and the problems still associated with infectious diseases is essentially reflected by facts. However, each Eastern European country is struggling under different conditions with similar problems, but this is also successful in many areas. Ukraine and Belarus, as two post-soviet (but not EU member) countries, are presented in the *Daily Mail* in a very similar way. More attention is paid to Ukraine, but the major topics are common: communicable diseases, lifestyle and alcohol consumption, life expectancy gender gap and peculiarities of maternal and children’s health. Despite the image created by the media, the real situation with public health in these two countries has both similarities and differences. Belarus and Ukraine are straggling with a significant life expectancy gender gap, rather high male mortality rates in productive age and high levels of alcohol consumption *per capita*. However, there are some substantial differences. Belarus achieved much better results in mortality rates and in reducing incidence rates of social diseases such as TB, HIV/AIDS and measles and also boasts low maternal death rate.

An example of the relationship between health and politics is the decision of the UK to leave the European Union. Combined demographic, health and financial reasons are one of the three ignition points of Brexit. The changes it will lead to, both in the UK and across the world, are yet unknown [30,89]. In our study, throughout the analysed period, there were publications presenting various points of view on health and medical care, but the texts from 2014–2017 showed the greatest aversion to immigrants. It can be concluded that the narrative in the analysed media articles published in those years contained a clearly greater number of politically engaged texts. Their message was based more on the beliefs and views of the authors than on hard data. They widely used BZSG, and the anomy affected everyone, the “hosts” and the “guests” alike. No conciliatory solutions were proposed in the news.

As for the unflattering epithets, they all appeared in different, more or less implicit contexts. In the texts (mostly on the subject of maternity) that put Eastern European women from EU countries together with overseas immigrants, one comes across expressions like “Eastern Europeans are still ‘not-quite-white’”. The whole subject of dispute on the EE members of the EU points to the label of “second class EU citizens”. However, it is difficult to confirm or rule out the use of the label “East European yet not fully European” based on the analysed texts. At the same time, it is worth emphasizing that all these labels are inappropriate in the context of the diverse community of contemporary Europeans.

The conducted analysis has a practical dimension not only in relation to the health aspect, but it also contains a certain warning to other EU countries as to what may result from (whether desired or not) manipulating public opinion.

## 7. Limitations

Discourse researchers believe that, critically, a completely impartial, neutral research perspective is impossible. The researcher may be involved in the subject or object of research in various ways [90,91]. For example, it is known that the statements of the national and ethnic majority and minorities are not equally assessed (the latter are often considered less reliable). The authors, being representatives of countries critically assessed by the British media, probably cannot remain completely neutral in this study.

## Data Availability

Not applicable.

## Appendix A

**Table A1:** FRAMES and Keywords in UK Tabloid Daily Mail Headlines and Text, 2006–2020.

No.	Country/Region	Title/Date of Publication/Position in References […]/Author	Frame: Keywords	Data on Which the Author Was Based
1	Ukraine	Woman, 22, shares candid selfies of severe acne after her skin suddenly broke out across her entire face “because of the stress of moving to another country”. (21 January 2020) [92] by V. Chalmers health reporter for Mailonline	D *: stress	American Academy of Dermatology, British Skin Foundation spokesperson
2	Romania	Parts of northern England have higher death rates than Turkey and Romania and the income divide between there and the south east is growing wider, report shows. (27 November 2019). [93] by J. Carr for Mailonline	D: death rates	The ‘State of the North 2019’ report, from IPPR North think tank
3	Poland Belarus	Mothers in the UK are almost FOUR TIMES more likely to die in childbirth than those from Poland and Belarus, UNICEF report reveals. (19 September 2019) [94] by S. Blanchard senior health reporter for Mailonline	C *: mothers	UNICEF
4	Czech Republic UK	Health chiefs warn children will die if measles isn’t stopped as they reveal four European countries including the UK have been stripped of their measles-free status in the last year. (29 August 2019) [95] by S. Blanchard senior health reporter for Mailonline	A *: measles	WHO European region
5	Ukraine UK	More than one in 10 children all over the world miss out on life-saving vaccines: Officials warn nearly 20 million are unprotected against measles, diphtheria and tetanus. (15 July 2019) [96] by A. Thompson senior health reporter for Mailonline	A: measles	The World Health Organization (WHO) and UNICEF
6	Ukraine Romania	Measles outbreak in Europe on track to eclipse last year’s toll as official figures show 34,000 have already been struck down by the killer infection in the first two months of 2019. (7 May 2019) [97] by S. Blanchard senior health reporter for Mailonline	A: measles	The World Health Organization (WHO)
7	Poland Ukraine	Berlin, Warsaw and other urban cities could be struck down by an evolved outbreak of measles ‘because of immigration mixed with low vaccination rates’. (22 January 2019) [98] by V. Chalmers health reporter for Mailonline	A: measles	The Jagiellonian University Medical College team
8	Italy, Germany and Romania	Measles epidemic warning: Deadly infection strikes more than 100 people across five regions as experts urge parents to vaccinate their children. (17 January 2018) [99] by A. Thompson health reporter for Mailonline	A: measles	NHS immunisation statistics
9	Ukraine among others	Women really ARE the stronger sex! They survive better than men during famines, epidemics and slavery, reveals analysis of some of humanities most “horrific” situations. (8 January 2018) [100] by C. Fernandez science correspondent for the Daily Mail	D: women	The researchers from the University of Southern Denmark
10	Eastern Europe	Record rates of HIV in Eastern Europe prompts health bosses to warn more must be done to combat the “unacceptably high” cases. (27 November 2018) [101] by S. Blanchard senior health reporter for Mailonline	A: HIV	The World Health Organization (WHO)
11	Many countries Russia, Belarus, Ukraine	Death map of the world: Major report of 180 countries reveals how many people will die before their 70th birthday (so, how does your nation rank?) (21 September 2018) [102] by S. Matthews assistant health editor for Mailonline	D: death rates	The report, published in the Lancet, Imperial College London scientists
12	Romania	Two care workers lied about a dementia patient’s cut mouth after she fell out of bed—claiming her injuries were caused by her FALSE teeth falling out. (8 August 2018) [103] by D. White for Mailonline	B *: health care workers	story
13	World	Is YOUR country prepared for a disease epidemic? Interactive map reveals only 14 per cent could cope, with Australia and South Korea at the top of the list. (22 June 2018) [104] by S. Blanchard senior health reporter for Mailonline	A: epidemic	PreventEpidemics.org
14	1.4 million Eastern Europeans living in Britain as a potential cause of problems Mainly Poland	Immigration is placing a strain on the NHS: Report reveals children born to Eastern European mothers has fuelled a 14% rise in intensive care admissions. (27 October 2017) [105] by S. Matthews for Mailonline	B:pressure on health care resources, C: children, A: AIDS, hepatitis B and C and TB	1.undefined reports 2. Paediatric Intensive Care Audit Network for children up to the age of 15 between 2004 and 2013. 2. Migrationwatch UK think tank.
15	Poland	Eight-months-pregnant British woman is refused treatment by Cambridge hospital unless she can prove she is English—after taking her Polish husband’s surname. (21 October 2017) [106] by J. Sheppard for Mailonline	B:pressure on health care resources, woman	Interview
16	Poland	Amid doctors’ hunger strike, Poland may boost health budget. (19 October 2017) [107] by Associated Press	B: health care	Reporters materials
17	Poland	Poland doctors rally for higher pay, more health care funds. (14 October 2017) [108] by Associated Press	B: health care	Reporters materials
18	Europe Romania	Alarm as measles spreads across Europe as countries struggle to vaccinate young children (28 March 2017) [109] by S. Matthews for Mailonline and AFP	A: measles, vaccination	The World Health Organization
19	African women; Poland	Health tourism ‘chaos’ draining the NHS: MPs condemn ministers over millions lost on foreign patients who don’t pay. (1 February 2017) [110] by S. Borland health editor for the Daily Mail	B: health tourism	Whitehall research
20	Eastern European immigrants	Show your passport if you want hospital treatment: Health mandarin’s proposal for ALL patients to curb health tourism. (12 December 2016) [111] by D. Martin chief political correspondent for the Daily Mail	B: pressure on health care resources	Politicians
21	A young man with Australian and Polish citizenship born in Australia	My shocking encounters with health tourists who exploit NHS. (23 November 2016) [112] by S. Reid for the Daily Mail	B: health tourism	Reporter private experience
22	Immigrants	Proposals to show passports for NHS treatment “go too far”. (22 November 2016) [113] by Press Association	B: pressure on health care resources	Politicians
23	Ukraine	US maternal mortality rate is worse than Iran and Ukraine with 27% spike in deaths since 2000. (10 August 2016) [114] by M. De Graaf for Dailymail.com	C: mothers	Centers for Disease Control and Prevention
24	2.5 million Romanians working abroad	Romania seeks to help children whose parents work abroad (21 July 2016) [115] by Associated Press	B: pressure on health care resources	The Romanian National Statistics Institute
25	East European members of EU	Rising social progress in East Europe likely to lead to drop in migration—economist. (29 June 2016) [116] by Reuters	D: healthy style of life	Thomson Reuters Foundation
26	Russia, Ukraine	HIV epidemic sweeps Europe with record number of cases reported—as WHO warns heterosexual sex and drug use is to blame. (27 November 2015) [117] by L. Parry for Dailymail.com	A: HIV	European Centre for Disease Prevention and Control (ECDC) and the WHO Regional Office for Europe
27	Romanians, Poles, Lithuanians and Slovakians	Scandal of online tips to “screw system”: Health tourists use forums to boast of milking NHS in their own countries. (9 August 2015) [118] by P. bentley, deputy investigations editor for the Daily Mail	B: pressure on health care resources	Tips uncovered by The Daily Mail on foreign language forums and blogs
28	Eastern Europeans	Ministers order urgent investigation into “completely unacceptable” revelations foreigners are charging the NHS for care in their OWN country (7 September 2015) [119] by P. Bentley, K. Faulkner and S. Borland for the Daily Mail investigations unit and T. Mctague, deputy political editor for Mailonline	B: pressure on health care resources	Politicians
29	Poland and Belarus	Why ARE women in the UK twice as likely to die in childbirth as their Polish peers? Expert blames a rise in obesity and older mothers. (7 May 2015) [120] by H. Cheyne for the Conversation	C: mothers	Save the Children’s 16th State of the World’s Mothers
30	23 countries: Argentina, Australia, Belgium, Brazil, Canada, China, France, Germany, India, Indonesia, Italy, Japan, Mexico, Poland, Russia, South Africa, South Korea, Spain, Sweden, Turkey, UK, Ukraine and the USA.	How healthy is YOUR country? Indonesians get the most sleep, the Japanese are the most unhealthy eaters, while Mexicans are the fittest. (6 January 2015) [121] by M. Davies for Mailonline	D: healthy style of life	Research organisation GfK
31	Poland	Paramedics recruited from Poland: Ambulance bosses forced to look abroad amid nationwide shortage of 3000 staff (24 December 2014) [122] by T. Kelly for the Daily Mail	B: pressure on health care resources	Reporters information
32	Poland	The English hospitals that need more nurses... but only if they can speak to their patients in Polish. (22 November 2014) [123] by M. Beckford, J. Macfarlane for the Mail on Sunday	B: pressure on health care resources	Reporters view
33	Romania	Romanian scrap worker pledges to bring his wife and five children to join him in UK “because it’s my right to £25,000 benefits and a four bedroom house”. (15 November 2014). [124] by C. Charlton for Mailonline	B: pressure on health care resources	Reporters view
34	Europeans	More than three million deaths worldwide are caused by alcohol each year—and Europeans drink more than anyone else. (13 May 2014) [125] by E. Innes	D: alcohol	World Health Organization (WHO)
35	Poland	Lifestyle “is better in Poland than Britain”: Less crime and violence—and it’s cheaper too. (29 September 2011) [126] by S. Poulter for the Daily Mail	D: healthy style of life	European Institute for Crime Prevention and Control some chosen data (645 comments)
36	Poland, India, Sri Lanka, Somalia, Afganistan, Pakistan	The NHS hospital where 80 per cent of babies have foreign mothers (1 May 2011) [127] by J. Macfarlane for Mailonline	B: pressure on health care resources	The statistics—released following a Freedom of Information request by The Mail on Sunday
37	Poland	5000 Polish babies born in UK every year. (2 December 2006) [128] by J. Lewis	C: baby boom	Lack of source of data

* A—Diseases; * B—Health care resources; * C—Maternity; * D—Style of life; [position in References].
